# Extracellular aminopeptidase regulates exopolysaccharide production of *Pseudomonas aeruginosa* via quorum sensing

**DOI:** 10.1093/ismejo/wraf038

**Published:** 2025-03-02

**Authors:** Tianhu Zhao, Fanglin Lei, Zhenyu Zhang, Di Wang, Luyan Z Ma

**Affiliations:** State Key Laboratory of Microbial Resources, Institute of Microbiology, Chinese Academy of Sciences, Beijing 100101, China; College of Life Sciences, University of Chinese Academy of Sciences, No. 1 Yanqihu East Rd, Huairou District, Beijing 101408, PR China; State Key Laboratory of Microbial Resources, Institute of Microbiology, Chinese Academy of Sciences, Beijing 100101, China; School of Life Sciences, Yunnan University, Kunming 650091, China; State Key Laboratory of Microbial Resources, Institute of Microbiology, Chinese Academy of Sciences, Beijing 100101, China; College of Life Sciences, University of Chinese Academy of Sciences, No. 1 Yanqihu East Rd, Huairou District, Beijing 101408, PR China; State Key Laboratory of Microbial Resources, Institute of Microbiology, Chinese Academy of Sciences, Beijing 100101, China; State Key Laboratory of Microbial Resources, Institute of Microbiology, Chinese Academy of Sciences, Beijing 100101, China; Medical School, University of Chinese Academy of Sciences, No. 1 Yanqihu East Rd, Huairou District, Beijing 101408, PR China; College of Life Sciences, University of Chinese Academy of Sciences, No. 1 Yanqihu East Rd, Huairou District, Beijing 101408, PR China

**Keywords:** *Pseudomonas aeruginosa*, biofilm, aminopeptidase, exopolysaccharide Psl, quorum sensing

## Abstract

The biofilm matrix primarily consists of proteins, exopolysaccharides, and extracellular DNA. *Pseudomonas aeruginosa* aminopeptidase is one of the most abundant matrix proteins in *P. aeruginosa* biofilms and plays a crucial role in modulating biofilm development. In a previous study, we have revealed that the loss of aminopeptidase enhances the attachment ability of *P. aeruginosa*. However, the mechanism by which aminopeptidase affects attachment remains unclear. In this study, we demonstrate that aminopeptidase is the primary protein associated with the matrix exopolysaccharide Psl. The loss of aminopeptidase leads to increased production of Psl, resulting in enhanced attachment of *P. aeruginosa.* Further investigation shows that aminopeptidase represses the transcription of the *psl* operon through the LasI/LasR quorum-sensing system, rather than via other known *psl* regulators or the cyclic-di-GMP signaling molecule. Aminopeptidase inhibits the transcription of *lasI* via the short peptides cleaved from the proform of aminopeptidase during its activation, which results in reduced biosynthesis of the quorum-sensing signaling molecule, *N*-(3-oxododecanoyl)-L-homoserine lactone, further decreasing the production of Psl. In conclusion, our study reveals an interplay between two key matrix components via quorum-sensing signal, suggesting a mechanism by which bacteria control initial attachment and exopolysaccharide production in response to cell density.

## Introduction

In natural environments, bacteria predominantly exist within biofilms, wherein bacterial cells are encased in a self-secreted extracellular matrix. The biofilm matrix typically comprises extracellular proteins, exopolysaccharides, and extracellular DNA (eDNA), which collectively maintain the structural integrity of the biofilm and facilitate bacterial survival under harsh environmental conditions [1]. *Pseudomonas aeruginosa* is a model organism for biofilm research and recognized as an opportunistic pathogen, significantly contributing to infections in patients with cystic fibrosis (CF) and individuals with compromised immune systems [2]. *P. aeruginosa* is capable of forming biofilms on both biotic and abiotic surfaces. One of the most abundant extracellular proteins identified in the biofilm matrix of *P. aeruginosa* is aminopeptidase [3]. However, there is limited understanding regarding the potential cooperative regulatory mechanisms among matrix components, particularly at the transcriptional level.

Aminopeptidase is classified as an exopeptidase that catalyzes the hydrolysis of the peptide or protein substrates from the N-terminus and releases free amino acids [4]. *Pseudomonas aeruginosa* aminopeptidase (PaAP) belongs to M28 family, which preferentially cleaves both leucine and lysine-*p*-nitroanilide substrates [5, 6]. The gene locus of PaAP is PA2939, which encodes a protein consisting of 536-amino acids. It has a signal peptide at the N-terminus, followed by a short auto-cleaved fragment. PaAP is secreted as a precursor protein that requires cleavage at the C-terminus by a series of extracellular proteases for activation [7, 8]. Subsequently, an auto-processing at the N-terminus cleavage site completes the activation process [5]. The active site residues of PaAP have been previously predicted, including the amino acid residues His-296, Asp-308, Glu-341, Asp-369, and His-467 [5]. PaAP not only exists extracellularly as a soluble protein, but also is the major protein associated with outer membrane vesicles (OMVs) [9, 10]. Moreover, PaAP has been reported to participate in interactions with lung epithelial cells [10]. The expression of PaAP significantly increases when *P. aeruginosa* is cultured on host epithelial cells, indicating PaAP's important role in responding to bacteria–host interaction [11].

PaAP expression is regulated by the stress response sigma factor RpoS and the quorum-sensing (QS) system [12, 13]. QS is the most common way of information exchange among microorganisms. Bacteria within the biofilm matrix communicate with each other through secreted QS signaling molecules. *P. aeruginosa* utilizes four distinct QS signaling systems, namely Las, Rhl, Pqs, and Iqs systems [14–17], among which the Las and Rhl systems are acyl homoserine lactone (AHL) QS circuits. LasI is required for the synthesis of the QS signaling molecule, *N*-(3-oxododecanoyl)-L-homoserine lactone (C12-HSL), whereas LasR acts as a C12-HSL-dependent transcriptional activator. LasR activates the transcription of *lasI*, *rhlI*, and *rhlR*. RhlI synthesizes the diffusible QS signaling molecule, *N*-butyryl-HSL (C4-HSL), and RhlR is a C4-HSL-dependent transcriptional activator [18]. LasR and RhlR regulate a wide range of genes in *P. aeruginosa*, including those related to exopolysaccharide production and virulence. PaAP has also been reported to be regulated by the Las QS system, which relies on C12-HSLs as signaling molecules. At high cell density, PaAP expression increases by over 30-fold [13].

PaAP has been reported to limit the early biofilm formation of *P. aeruginosa* [28]. Loss of PaAP in *P. aeruginosa* leads to enhanced attachment and early stages of biofilm formation [20]. However, the mechanism by which PaAP affects attachment and early stages of biofilm formation remains unclear. Multiple factors affect the attachment ability of *P. aeruginosa*, including surface appendages such as flagella and type IV pili, as well as exopolysaccharides like Psl [22, 29–31].

Psl is critical for the initial attachment of *P. aeruginosa* to both biotic and abiotic substrates [22, 32]. It plays a key role in biofilm scaffolding and maintaining biofilm structure [33]. Psl can either be associated with the bacterial cell surface or released into the extracellular matrix [34]. Psl interacts with eDNA to form an eDNA–Psl fiber matrix, which functions as a skeleton of biofilm [35]. The genes responsible for synthesizing Psl exopolysaccharides are located within a 12-gene operon [36]. The alternative sigma factor RpoS and the QS regulator LasR positively regulate the transcription of the *psl* operon, whereas the transcriptional regulator AmrZ represses its transcription [36–38]. Moreover, the intracellular level of the second messenger cyclic-di-GMP (c-di-GMP) also affects Psl production and biofilm formation [39].

In this study, we have demonstrated how PaAP regulates the attachment and the production of the exopolysaccharide Psl in *P. aeruginosa*. The evidence that PaAP is closely associated with exopolysaccharide Psl suggests a potential correlation between these two key components of the biofilm matrix. Further investigation reveals that PaAP inhibits the biosynthesis of the exopolysaccharide Psl through the QS system, thereby weakens the bacteria’s ability to attach on surfaces. These findings provide a comprehensive understanding of the interactions among extracellular proteins, exopolysaccharides, and the QS system, which collectively guide the bacteria in initiating attachment in response to varying cell densities.

## Materials and methods

### Bacterial strains and growth conditions

Bacterial strains and plasmids used in this work were listed in Table 1. Plasmids were constructed using standard molecular cloning techniques. *P. aeruginosa* strains were grown at 37°C in Luria-Bertani medium without sodium chloride (LBNS), Jensen’s medium [40], M63 minimal medium [41], or an artificial sputum medium (ASM) [42]. Antibiotics were added to the media at the following final concentrations: 300 μg/ml carbenicillin, 30 μg/ml gentamycin, and 100 μg/ml tetracycline for *P. aeruginosa*. Artificially synthesized peptides (Beijing SciLight Biotechnology Ltd, Co.) were dissolved in ddH_2_O and applied to the bacterial growth culture when required.

**Table 1:** Strains and plasmids used in this study.

Strain or plasmid	Genotype and/or relevant characteristics	Source or reference
*P. aeruginosa* strains		
*P. aeruginosa* PAO1	Prototroph	[19]
Δ*paaP*	In-frame deletion of *paaP*(PA2939)	[20]
*paaP*::Tn5	*paaP* Tn5 insertion mutant, Tc^r^	[21]
Δ*paaP*::*paaP*	inserting *paaP* gene along with its promoter region into the *attB* site on chromosome of Δ*paaP*	[20]
WFPA800 (ΔP*_psl_*)	Psl-negative strain, *psl* operon promoter deletion mutant, ΔP*_psl_*	[22]
WFPA801 (P_BAD_-*psl*)	Psl-overproduced strain, P_BAD_-*psl*	[22]
ΔP*_psl_* Δ*paaP*	*psl* operon promoter deletion in Δ*paaP*	This study
PAO1/ vector	PAO1 carrying pHerd20T	[20]
PAO1/ pLasI	PAO1 carrying pHerd20T with *lasI*, Apr	This study
Δ*paaP*/ vector	Δ*paaP* carrying pHerd20T	[20]
Δ*paaP*/ pPaAP	Δ*paaP* carrying pHerd20T with *paaP*, Ap^r^	[20]
Δ*paaP*/ pD308A	Δ*paaP* carrying pHerd20T with PaAP^D308A^, Ap^r^	[20]
Δ*paaP*/ pPaAPNS	Δ*paaP* carrying pHerd20T with signal peptide truncated *paaP*, Ap^r^	[20]
Δ*lasI*	In-frame deletion of *lasI* in PAO1	This study
Δ*paaP*Δ*lasI*	In-frame deletion of *lasI* in Δ*paaP*	This study
Δ*lasI*/ pLasI	Δ*lasI* carrying pUCP20 with *lasI*, Ap^r^	This study
Δ*paaP*Δ*lasI*/ pLasI	Δ*paaP*Δ*lasI* carrying pUCP20 with *lasI*, Ap^r^	This study
Δ*paaP*/ pLasI	Δ*paaP* carrying pHerd20T with *lasI*, Apr	This study
Δ*lasR*	In-frame deletion of *lasR* in PAO1	This study
Δ*paaP*Δ*lasR*	In-frame deletion of *lasR* in Δ*paaP*	This study
Δ*lasI*Δ*rhlI*/ pPaAP	Δ*lasI*Δ*rhlI* carrying pHerd20T with *paaP,* Ap^r^	This study
Plasmids		
pHerd20T	*Escherichia coli-P. aeruginosa* shuttle plasmid containing arabinose inducible P_BAD_ promoter, Ap^r^	[23]
mini-CTX *lacZ*	*lacZ* transcriptional fusion, *attB* integration construction vector, Tc^r^	[24]
pFLP2	FLP recombinase expressing plasmid, Ap^r^	[25]
pPROBE-AT’	Promoter probe vector carrying *gfp* reporter, Ap^r^	[26]
p*psl*:*gfp*	pPROBE-AT’ carrying P*psl*::*gfp* fusion	This study
p*lasI:*:*gfp*	pPROBE-AT’ carrying p*lasI*::*gfp* fusion	This study
pTH2	mini-CTX *lacZ* containing p*lasI*::*lacZ* fusion, Tc^r^	This study
pTH3	mini-CTX *lacZ* containing P*algC*-*lacZ* fusion, Tc^r^	This study
pTH9	pPROBE-AT’ containing *psl* operon promoter-*gfp* fusion, Ap^r^	This study
pCdrA::*gfp* (ASV)^s^	pUCP22Not-PcdrA-RBS-CDS-RNaseIII-*gfp* (ASV)-T0-T1, Ap^r^ Gm^r^	[27]
pAlgC-FLAG	pHerd20T with arabinose inducible *algC* with FLAG-tag at C-terminal, Ap^r^	This study

### Rapid attachment assay in microtiter dish

Rapid attachment assays were performed as previously described with modifications [22, 43]. One-hundred-microliter of mid-log phase culture (OD_600_ ~ 0.5) grown in Jensen’s medium was inoculated in microtiter dish (BD Falcon) and incubated at 30°C for 30 min statically. The surface-attached cells were stained with 0.1% crystal violet, which was subsequently solubilized in 30% acetic acid for the OD_560_ measurement. To induce transcription of *paaP* from the pBAD promoter in pHerd20T vector, 1% arabinose was added to Jensen’s medium.

### Aminopeptidase assay

The PaAP catalytic activity was determined as previously described [7]. The supernatant of *P. aeruginosa* LBNS culture was collected for determining the PaAP catalytic activity. Equal volumes of substrate Leu-*p*-nitroanilide solution (1.2 mM Leu-*p*-nitroanilide in 0.1 M Tris–HCl, 2 mM CaCl_2_, pH 8.3) were added and incubated at 50°C for 15 min. A molar extinction coefficient of 10 400 for *p*-nitroaniline production from nitroanilide-based substrates was used to define the PaAP catalytic activity, calculated in nmol of nitroanilide per OD_600_ of the corresponding original culture.

### Detection of gene transcription

*P. aeruginosa* strains containing the gene promoter-*gfp* fusion plasmid were grown at 30°C with shaking. The green fluorescence intensity of the culture was measured using a Synergy H4 Hydrid Reader (BioTek). The transcription level of the genes to be tested was normalized to the biomass (OD_600_) of corresponding culture.

### β-Galactosidase assay

β-Galactosidase activity was determined as previously described [44]. *P. aeruginosa* strains were grown in Jensen’s medium with shaking at 200 rpm at 37°C to an OD_600_ of 0.5–0.8. Bacterial pellet from 2-ml culture aliquots were resuspended in 200 μl of Z-buffer and frozen/thawed three times to lyse the cells. The cell lysates were determined for both β-galactosidase activities and total proteins in BCA assay (Pierce, USA). The unit of β-galactosidase was defined as 1 μM of substrate hydrolyzed per min per mg/ml protein.

### Relative quantitative real-time PCR

Bacteria were grown approximately to an OD_600_ of 0.5, then total RNA was extracted using Trizol according to manufacturer’s specifications (Takara Bio Inc., Japan). Genomic DNA was removed by RNase-Free DNase I (New England Biolabs, Ltd) for 30 min at 37°C, and the RNA was purified using RNA clean Kit (TIANGEN Biotech Co., Ltd). The total DNase-treated RNA (10 μg) was reversely transcribed to synthesize cDNA using the M-MLV Reverse Transcriptase (Promega Co., Ltd) with random hexamer primers according to the manufacturer’s protocol. SYBR Green Mix (Roche) was applied in relative quantitative real-time PCR system in LightCycler 480 (F. Hoffmann-La Roche, Ltd). The expression of the selected genes was normalized using *rpsL* as an internal control. Relative expression levels of *amrZ*, *rsmA*, *rpoS*, and *lasR* were calculated by the relative quantification method (ΔΔC_T_) as previously described [45]. All assays were performed in triplicates.

### Immunodot blotting and western blotting

Exopolysaccharide Psl extracts were collected from 10 OD_600_ of overnight cultures and purified according to a previously published method [46]. Extracellular polymeric substance (EPS) was further examined in immune-dot blotting using anti-Psl antibody as previously described [46]. For the effect of QS signaling molecule (C12-HSL) on Psl production, 5 μM of C12-HSL was added to the medium after inoculation [47]. The immunodot blotting result was quantified in gray value using Image Lab software.

To detect PaAP in EPS, the WFPA801 and WFPA800 strains were cultured on cellophane covered LBNS agar plates with 1% arabinose for 24 h, and then the EPS of WFPA801 and WFPA800 were obtained through treatment with DNase I and RNase as previously described [46], but without Proteinase K treatment. The EPS (10 μg) was dissolved in SDS-PAGE loading buffer and loaded onto SDS-PAGE gel, transferred to nitrocellulose membrane, and then incubated with anti-PaAP antibody (1:5000) [7]. The protein bands detected in EPS were subjected to mass spectrometry analysis. Briefly, gel pieces containing the protein bands were subjected to reduction and alkylation using iodoacetamide and then digested with trypsin. Extracted peptides were analyzed by nano electrospray ionization mass spectrometry on Bruker Esquire HCT ion trap mass spectrometer. Mass spectra were searched against available databases using Mascot. To detect PaAP in the culture supernatant, an overnight culture was diluted 100-fold in Jensen’s medium and incubated for 24 h. The extracellular protein in the culture supernatant was precipitated with 100% TCA at a final concentration of 15%. The samples were dissolved in SDS-PAGE loading buffer and detected by western blotting using an anti-PaAP antibody.

To detect FLAG-tagged AlgC, approximately 10 OD bacteria were dissolved in SDS-PAGE loading buffer and detected using anti-FLAG antibody (1:5000) (Abmart, Shanghai, China).

### Detection of intracellular cyclic-di-GMP levels

Plasmid pCdrA::*gfp* (ASV)^s^ was used to detect c-di-GMP [27]. The green fluorescence intensity was measured in a Synergy H4 Hydrid Reader (BioTek), and normalized to absorbance at 600 nm.

### Collection and detection of short peptides in the supernatant

The overnight culture of the Δ*lasI*Δ*rhlI*/pPaAP strain was inoculated at 1:100 into Jensen’s medium with 1% arabinose, and incubated at 37°C and 200 rpm for 24 h. Cell culture was centrifuged at 12 000 rpm for 30 min at 4°C. The supernatant was added with three times the volume of ethyl acetate. The ethyl acetate layer (upper layer) was collected. After evaporation of ethyl acetate, the remaining precipitate was dissolved in methanol. Three times the volume of hexane was added for degreasing and the lower layer of liquid was collected. An equal volume of H_2_O and twice the volume of dichloromethane were added to the sample and mixed, and the dichloromethane layer (lower layer) was collected. After a complete evaporation of dichloromethane in the fume hood, chromatography grade methanol was added to dissolve the remaining precipitate. To remove pigments from the sample, a C18 reverse solid-phase extraction column was applied. After evaporation of methanol, the short peptides in the sample were detected by mass spectrometry.

## Results

### *Pseudomonas aeruginosa* aminopeptidase is the primary protein associated with the exopolysaccharide Psl matrix

**Figure 1:**
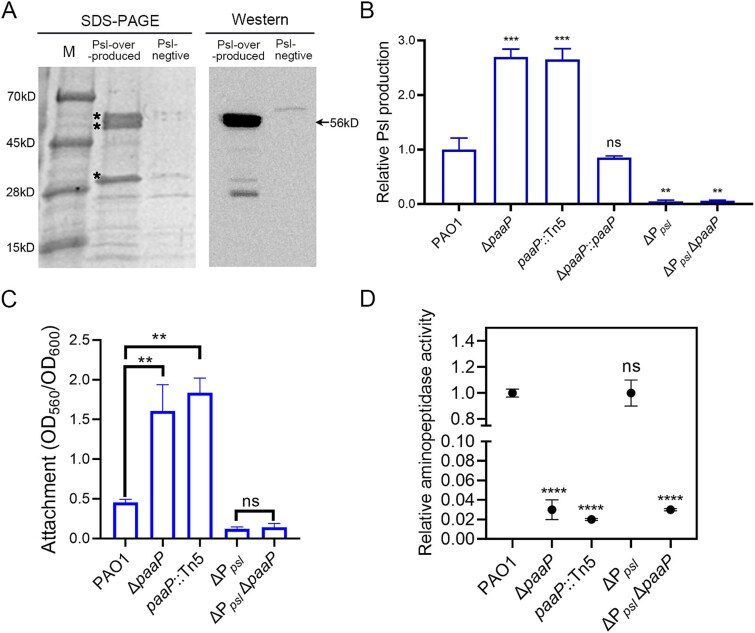
PaAP is associated with the Psl exopolysaccharide, and loss of PaAP enhanced initial attachment by increasing Psl production in *P. aeruginosa*. (A) Detection of proteins co-purified with EPS prepared from the Psl-overproducing strain WFPA801 and the Psl-negative strain WFPA800. Left panel, Coomassie brilliant blue staining of EPS samples on an SDS-PAGE gel; right panel, the corresponding western blotting result of the EPS samples detected using an anti-PaAP antibody. ^*^, protein bands determined as PaAP by mass spectrometry. (B) The Psl production of *P. aeruginosa* wild-type strain PAO1, the PaAP in-frame deletion mutant Δ*paaP*, the PaAP Tn5 insertion mutant *paap*::Tn5, the complementary strain Δ*paaP*::*paaP*, Psl-negative strain ΔP*_psl_*, Psl and PaAP double deletion strain ΔP*_psl_*Δ*paaP*, relative to the amount of Psl of PAO1 (=35 μg/ml). (C) The initial attachment of each strain. (D) The relative aminopeptidase activity (AP activity) in the culture supernatant of each strain, normalized to that of PAO1 (=3.6 nmol nitroanilide/OD_600_). Strains were grown with 1% arabinose for P_BAD_ promoter induction. Experiments were carried out in triplicate. Error bars indicate standard deviations. Statistical significance compared to PAO1 (unless otherwise indicated) was determined by Student’s *t*-test, and error bars indicate standard deviations, ^*^^*^P < .01, ^*^^*^^*^P < .001, ^*^^*^^*^^*^P < .0001, ns, not significant.

To investigate whether Psl also interacts with extracellular proteins present in the biofilm matrix of *P. aeruginosa*, we extracted EPS from the bacterial lawn of the Psl-over-producing strain WFPA801 and the Psl-negative strain WFPA800 from LBNS agar plates supplemented with 1% arabinose. The EPS was analyzed using SDS-PAGE. Three major protein bands were detected (Fig. 1A; two bands were approximately 56 kDa, one was approximately 28 kDa) in the EPS of WFPA801. Mass spectrometry analysis revealed that these three bands corresponded to PaAP, which was further confirmed by western blotting using an anti-PaAP antibody (Fig. 1A). The two high-molecular-weight bands (~56 kDa) were also identified in the EPS extracted from the wild-type strain PAO1 through Coomassie brilliant blue staining and anti-PaAP western blotting (data not shown). In contrast, little PaAP was detected in the EPS of the Psl-negative strain WFPA800 (ΔP*_psl_*), although several detectable protein bands were observed in the SDS-PAGE gel (Fig. 1A). These results indicate that PaAP is the primary protein enriched in the Psl matrix, suggesting an association between PaAP and the Psl matrix in biofilms.

### Loss of *Pseudomonas aeruginosa* aminopeptidase resulted in increased production of Psl

The Δ*paaP* mutant has been reported to exhibit enhanced ability of attachment compared to the wild-type strain PAO1 [20]. Psl, flagella, and type IV pili all affect the initial attachment of *P. aeruginosa* [22, 29, 30]*.* The Δ*paaP* mutant displayed wild-type-levels of motility, indicating normal function of both the flagella and type IV pili [20]. As PaAP is associated with the Psl exopolysaccharide, as described above, we hypothesized that the absence of PaAP might lead to changes in Psl levels, which resulted in enhanced initial attachment of the Δ*paaP* mutant. In consistent with our hypothesis, the production of Psl was found to be 2 to 3 times higher in the Δ*paaP* mutant than in PAO1 (Fig. 1B). The phenotype of a Tn5 mutant with an insertion mutation in the *paaP* gene showed similar results as that in the Δ*paaP* in-frame deletion mutant. Moreover, Psl production in the Δ*paaP* mutant was restored to wild-type levels by expressing *paaP* inserted at the chromosomal *attB* site (Δ*paaP*::*paaP*) (Fig. 1B)*.* These data suggest that the enhanced surface adherence of the Δ*paaP* mutant is likely due to the increased Psl production. To further confirm this hypothesis, we constructed a double knockout mutant of Psl and PaAP (ΔP*_psl_*Δ*paaP*) by deleting the promoter region of the *psl* operon in the Δ*paaP* strain. The resulting ΔP*_psl_*Δ*paaP* mutant exhibited minimal attachment to the wells of the microtiter dish, similar to that of the Psl single knockout strain WFPA800 (ΔP*_psl_*) (Fig. 1C). This result indicates that the enhanced surface adherence of the Δ*paaP* mutant is dependent on Psl. In contrast, Psl production did not affect the catalytic activity of extracellular PaAP (Fig. 1D). Overall, our data demonstrate that the loss of PaAP enhances the initial attachment of *P. aeruginosa* by increasing Psl production, suggesting that the expression of PaAP inhibits Psl biosynthesis.

### An active extracellular *Pseudomonas aeruginosa* aminopeptidase is required to inhibit Psl production

**Figure 2:**
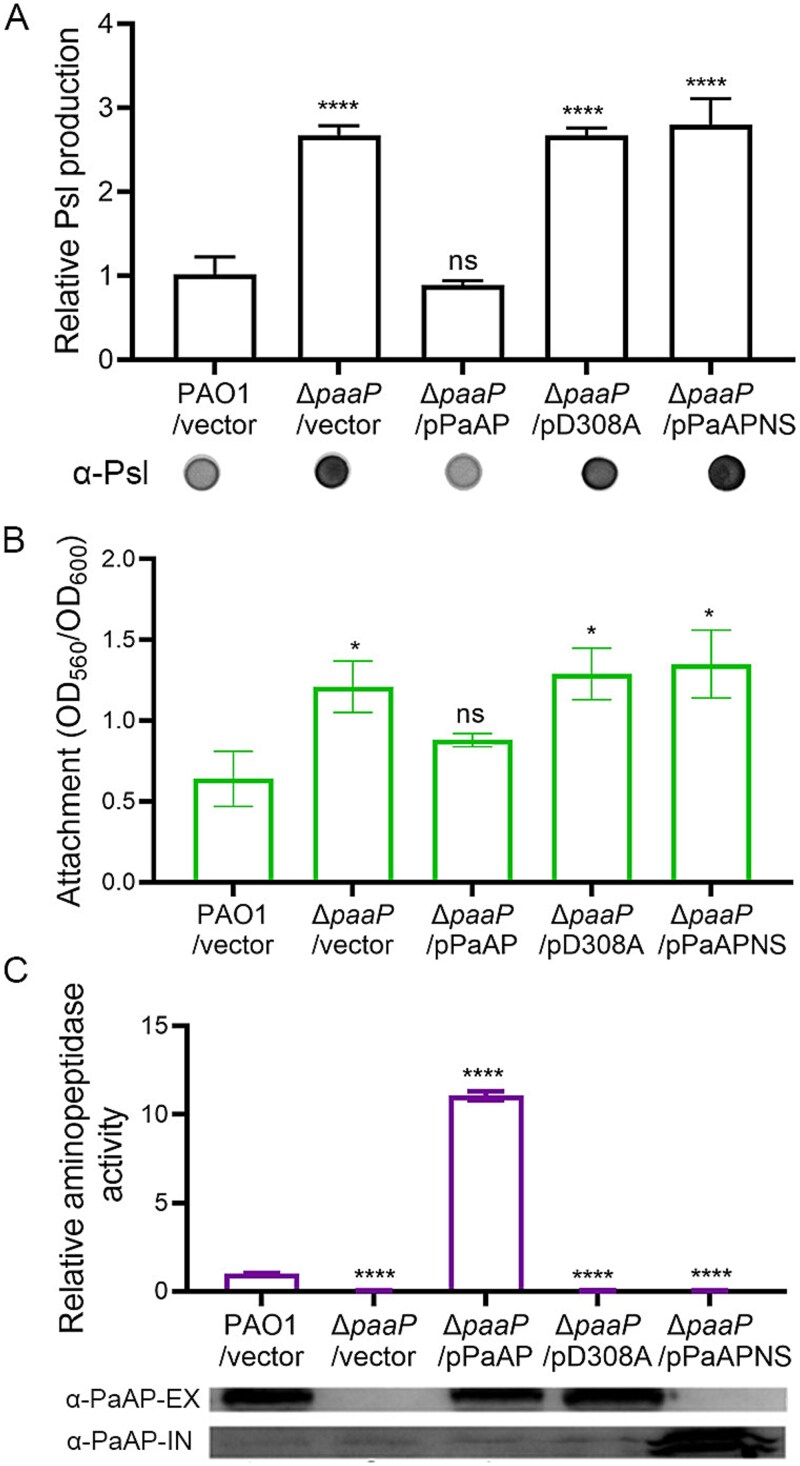
An active extracellular PaAP is required to inhibit Psl production. (A) The Psl production of Δ*paaP* with wild-type PaAP expressed from plasmid pHerd20T (the result of Δ*paaP/*pPaAP), the mutated PaAP (D308A) (the result of Δ*paaP/*pD308A) and the PaAP lacking the signal peptide (PaAPNS) (the result of Δ*paaP/*pPaAPNS). (B) The initial attachment of each strain. (C) The aminopeptidase activity (AP activity) of each strain normalized to that of PAO1/vector (AP =3.2 nmol nitroanilide/OD_600_), and the extracellular (α-PaAP-EX) and intracellular PaAP (α-PaAP-IN) in corresponding planktonic culture, detected by immunoblotting using an anti-PaAP antibody. Strains were grown with 1% arabinose for PBAD promoter induction. Experiments were carried out in triplicate. Error bars indicate standard deviations. Statistical significance compared to PAO1/vector was determined by Student’s *t*-test, and error bars indicate standard deviations, ^*^P < .05, ^*^^*^^*^^*^P < .0001, ns, not significant.

To further investigate whether the aminopeptidase catalytic activity of PaAP is necessary for modulating Psl production, we assessed the ability of the PaAP^D308A^ variant to complement Psl production in the Δ*paaP* mutant. The PaAP^D308A^ variant has the key catalytic amino acid, Asp308, mutated to alanine [5, 20]. The PaAP^D308A^ expressed from the plasmid pD308A completely lost its aminopeptidase catalytic activity and was unable to complement the Δ*paaP* phenotype, including Psl production and surface attachment (Fig. 2A and B). Additionally, the PaAP variant lacking the signal peptide (PaAPNS) also failed to complement the Δ*paaP* phenotype (Fig. 2A and B). In contrast, all phenotypes of the Δ*paaP* strain were successfully complemented by wild-type PaAP (Fig. 2A and B). Given that PaAP and its variants exhibited similar expression levels, as indicated by western blotting against an anti-PaAP antibody (Fig. 2C), these results suggest that PaAP functions extracellularly and its aminopeptidase catalytic activity is critical for inhibiting Psl production.

### *Pseudomonas aeruginosa* aminopeptidase inhibits Psl production at the transcriptional level

**Figure 3:**
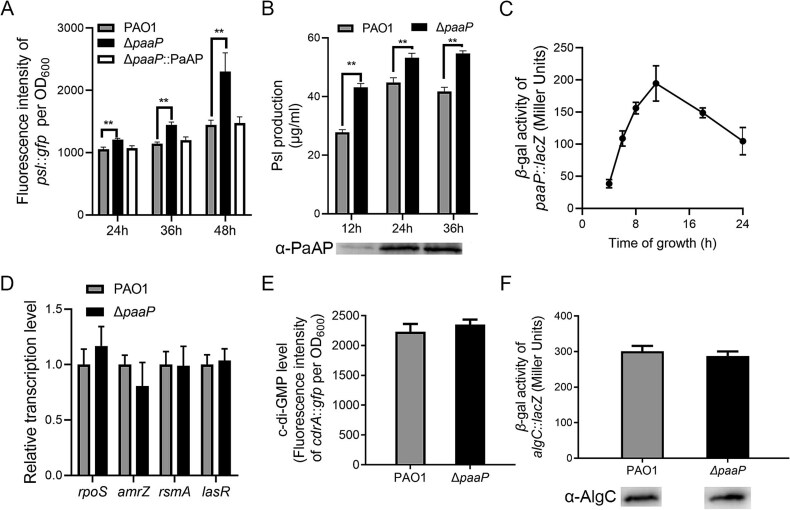
Deletion of PaAP induces the transcription of the *psl* operon without affecting the transcription of other Psl-regulator genes, the expression of AlgC, or the intracellular level of c-di-GMP. (A) Transcription of *psl* in the planktonic cultures of PAO1, Δ*paaP*, and Δ*paaP*::*paaP* after 24 h, 36 h, and 48 h of growth, detected by the *psl::gfp* reporter plasmid. (B) Psl production of PAO1 and Δ*paaP* after 12 h, 24 h, and 36 h of growth, and PaAP in PAO1 planktonic culture detected by immunoblotting at each time point. (C) The time course of *paaP* transcription in PAO1. (D) The relative transcription level of *psl* regulators: *rpoS*, *amrZ*, *rsmA*, and *lasR*, analyzed by relative quantitative real-time PCR (normalized to PAO1 level, respectively). (E) The intracellular level of the c-di-GMP in PAO1 and Δ*paaP* indicated by fluorescence intensity of plasmid pCdrA::*gfp* (ASV)^s^. (F) The level of *algC* transcription and AlgC expression in PAO1 and Δ*paaP* strains. Experiments were carried out in triplicate. Significance was determined by Student’s *t*-test, and error bars indicate standard deviations, ^*^^*^P < .01.

To investigate how PaAP affects the production of Psl, we examined the transcription of the *psl* operon in the Δ*paaP* mutant and the wild-type strain PAO1. Using a *psl*::*gfp* fusion, we found that *psl* transcription was significantly higher in Δ*paaP* compared to PAO1, which was complemented to wild-type levels in the complementary strain Δ*paaP*::*paaP* (Fig. 3A). Consistently, Psl production was higher in Δ*paaP* than that in PAO1 (Fig. 3B). This difference was observed in the logarithmic phase culture (12 h) as well as the stationary culture (24 h and 36 h in Fig. 3B). We further investigated the transcription of *paaP* (Fig. 3C) and the level of extracellular PaAP (Fig. 3B) at various time points. The results indicated that the transcription of *paaP* starts at early stage of the logarithmic phase and the PaAP protein accumulates as bacteria grow. Taken together, these results indicate that PaAP inhibits Psl production at the transcriptional level.

We then examined the transcription levels of four known Psl regulators, RpoS, AmrZ, RsmA, and LasR in the Δ*paaP* mutant (Fig. 3D) [37, 48, 49]. None of these four regulators exhibited transcriptional differences in the Δ*paaP* mutant compared to PAO1. As intracellular c-di-GMP levels also affects Psl production in *P. aeruginosa*, we then measured the intracellular c-di-GMP levels using pCdrA::*gfp* (ASV)^s^ as an indicator [39]. The Δ*paaP* mutant exhibited a fluorescence intensity similar to that of PAO1, suggesting comparable c-di-GMP levels (Fig. 3E). AlgC has been reported to provide the sugar precursors for Psl synthesis [46], however, the transcription of *algC* and the expression of AlgC showed no differences between Δ*paaP* and PAO1 (Fig. 3F). These data indicated that neither the four *psl* regulators, c-di-GMP nor AlgC was involved in the PaAP-mediated repression of *psl* transcription.

### *Pseudomonas aeruginosa* aminopeptidase inhibits Psl production through a C12-HSL-dependent quorum-sensing system

**Figure 4:**
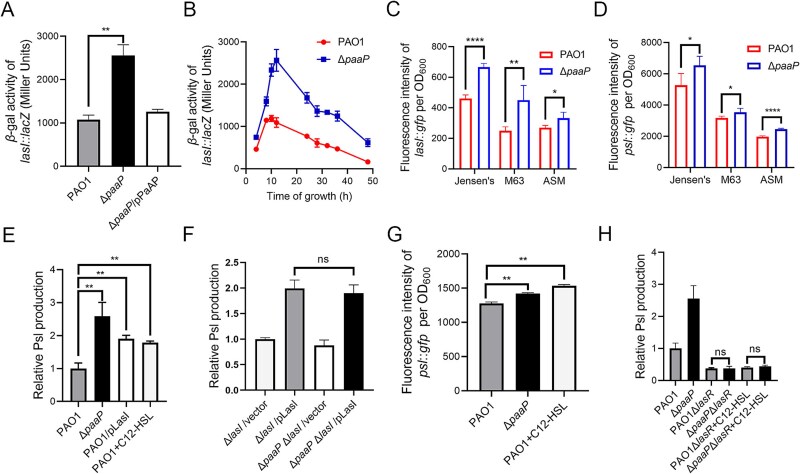
PaAP limits Psl production by inhibiting transcription of *lasI*. (A) The transcription of *lasI*::*lacZ* in PAO1, Δ*paaP*, and Δ*paaP/*pPaAP after 12 h of growth. (B) The time course of the transcription of *lasI* in PAO1and Δ*paaP* within 48 h. (C) The transcription of *lasI* and (D) the transcription of *psl* in PAO1 and Δ*paaP* in Jensen’s, M63 and ASM medium after 12 h of growth. (E) The relative Psl production of PAO1, Δ*paaP*, PAO1/pLasI, and PAO1 supplied with C12-HSL [47]. (F) The relative Psl production of the Δ*lasI*, Δ*paaP*Δ*lasI* strains, and their corresponding *lasI* complementary strains. (G) Transcription of *psl* in PAO1, Δ*paaP*, and PAO1 in the presence of 5 μM of C12-HSL detected using the *psl::gfp* reporter plasmid. (H) The relative Psl production of PAO1, Δ*paaP*, and Δ*lasR*, Δ*paaP*Δ*lasR* with or without C12-HSL. Experiments were carried out in triplicate. Significance was determined by Student’s *t*-test, and error bars indicate standard deviations, ^*^P < .05, ^*^^*^P < .01, ^*^^*^^*^^*^P < .0001; ns, not significant.

As we observed a significant difference in Psl production between the Δ*paaP* mutant and wild-type PAO1 during the late stages of bacterial growth (Fig. 3A and B), we investigated whether QS is involved in the regulation of Psl production by PaAP. We examined the transcription of *lasI*, which encodes the protein responsible for synthesizing QS signals and has been reported to affect biofilm formation and carbohydrates production in the biofilm matrix [50]. The transcription of *lasI* in Δ*paaP* was 3-fold higher than in PAO1, which could be complemented by PaAP expressed from a plasmid (Fig. 4A). The disparity in *lasI* transcription between Δ*paaP* and PAO1 emerged within 2 h of bacterial growth and lasted for over two days (Fig. 4B). As the deletion of PaAP did not impact bacterial growth [20], the increased *lasI* transcription in Δ*paaP* is not attributable to changes in cell densities. We have also assessed the transcription of *lasI* in Δ*paaP* compared to PAO1 in various media including M63 minimal medium and ASM, which simulates the nutrient conditions in the lungs [42]. In M63 and ASM media, the transcription levels of *lasI* and *psl* were both higher in Δ*paaP* than in PAO1 (Fig. 4C and D), indicating that PaAP inhibits the *lasI* transcription and the *psl* transcription under different nutritional conditions. However, the transcription levels of *lasI* and *psl* in either PAO1 or Δ*paaP* were lower in M63 and ASM media compared to those in Jensen’s medium, which may be attributed to the limited nutrient availability in these two media (Fig. 4D).

Expressing LasI in PAO1 also increased Psl production, similar to that observed in the Δ*paaP* mutant (Fig. 4E), suggesting that LasI may be involved in the inhibition of Psl production by PaAP. To test this hypothesis, we constructed Δ*lasI* and Δ*paaP* Δ*lasI* double mutants and introduced the plasmid pLasI, which constitutively expresses LasI to eliminate the influence of PaAP on LasI levels. The Δ*lasI/*vector and Δ*paaP*Δ*lasI*/vector mutants exhibited comparable Psl production, indicating that PaAP relies on LasI to regulate Psl (Fig. 4F). Furthermore, Psl production levels were similar in the Δ*lasI/*pLasI and Δ*paaP*Δ*lasI/*pLasI mutants (Fig. 4F), supporting the notion that PaAP controls Psl expression through LasI. LasI serves as the synthase for the QS signaling molecule, C12-HSL. Therefore, we examined whether the addition of C12-HSL to the growth media could influence Psl production in PAO1. Indeed, the addition of C12-HSL to the culture media increased Psl production (Fig. 4E), which was a result of the increase in the transcription of the *psl* operon (Fig. 4G). C12-HSL is typically associated with the transcriptional regulator LasR to activate their target genes. To determine whether the effect of C12-HSL on Psl production is dependent on LasR, we constructed the *lasR* in-frame deletion mutant Δ*lasR,* and the Δ*lasR*Δ*paaP* double deletion mutant. Both strains exhibited a decrease in Psl production that could not be restored by the addition of C12-HSL (Fig. 4G). This result suggests that C12-HSL requires LasR to induce the expression of Psl, consistent with previous reports that LasR is a transcriptional activator of the *psl* operon [48]. It is known that the LasI-LasR QS system activates the expression of the extracellular enzyme PaAP [13]. Our data demonstrate that PaAP, in turn, represses the transcription of *lasI*, leading to the inhibition of *psl* transcription and reduced Psl production.

### *Pseudomonas aeruginosa* aminopeptidase inhibits *lasI* expression by means of short peptides that are cleaved from pro-*Pseudomonas aeruginosa* aminopeptidase during its enzymatic activation

**Figure 5:**
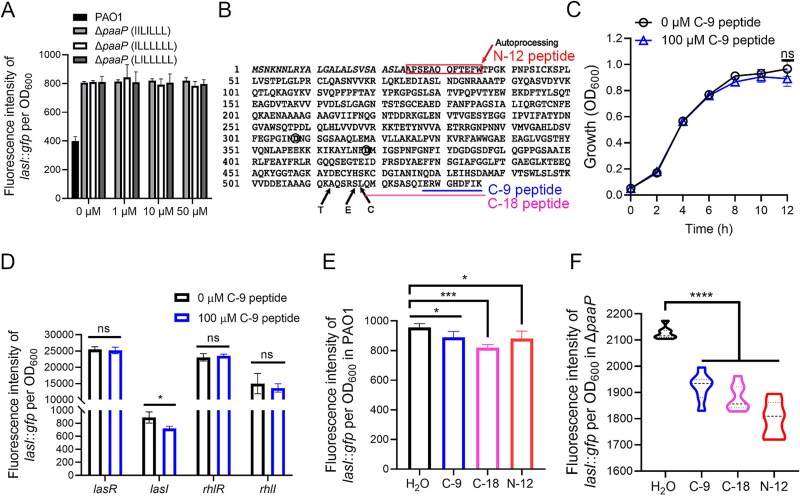
PaAP inhibits the transcription of *lasI* via short peptides cleaved from PaAP during PaAP activation. (A) Effect of short peptides from supernatant on *lasI* gene expression of Δ*paaP*. (B) Diagram of amino acid composition of PaAP [7]. T-trypsin, E-elastase, and C-chymotrypsin. (C) Growth curve of PAO1 with or without C-9 peptide supplement. (D) Effect of C-9 peptide on *lasI* transcription of PAO1. (E) and (F) Effect of 100 μM of C-9 peptide, C-18 peptide, and N-12 peptide on *lasI* transcription of PAO1 (E) and Δ*paaP* (F). The according volume of H_2_O was applied as control, as all the peptides were dissolved in H_2_O. Experiments were carried out in triplicate. Significance was determined by Student’s *t*-test, and error bars indicate standard deviations, ^*^P < .05, ^*^^*^^*^P < .001; ^*^^*^^*^^*^P < .0001, ns, not significant.

We then investigated how extracellular PaAP can regulate the transcription of the *lasI* gene. Extracellular peptides have been reported to function as signaling molecules that participate in the regulation of gene expression [51]. PaAP can remove amino acids from the N-terminus of proteins or oligopeptides, producing short peptides extracellularly. Therefore, we examined whether the short peptides generated by PaAP in the culture supernatant would participate in PaAP-mediated regulation of QS gene expression. To eliminate potential interference from QS signaling molecules, we constructed a *lasI* and *rhlI* double deletion strain in PAO1 and subsequently overexpressed PaAP under an inducible promoter, resulting in Δ*lasI*Δ*rhlI*/ pPaAP strain. Short peptides in the supernatant of the Δ*lasI*Δ*rhlI*/pPaAP strain were collected and identified using mass spectrometry. The sequences of the short peptides and their corresponding gene loci are presented in Table 2. The results indicated that the supernatant primarily contained short peptides rich in leucine and isoleucine, which aligns with the high catalytic efficiency of PaAP on these two amino acids. These short peptides were then artificially synthesized and applied to the Δ*paaP* strain to test their ability to repress the transcription of *lasI*. However, none of the synthesized short peptides were able to repress the transcription of *lasI* in the Δ*paaP* strain (Fig. 5A), suggesting that these short peptides may not be involved in the regulation of QS gene expression by PaAP.

**Table 2:** Short peptides rich in leucine and isoleucine in the supernatant and their corresponding coding gene loci.

Sequence	PA no.
ILLIILILLLI	PA3231
IILILLLI	PA3231
ILLLLLL	PA4002
LILLLLL	PA1360
IILILLL	PA3231
LILLLLL	PA1360

Previous studies have reported that PaAP is secreted out of the bacterial cells as an inactive form, and cleavage at its C-terminus can activate its peptidase activity [7]. During this enzymatic activation process, short peptides are produced from the cleavage of PaAP at its C-terminus or are auto-cleaved from its N-terminus (these peptides are referred to as C-18, C-9, and N-12 in this study) [7] (Fig. 5B). The analysis of the pro-PaAP crystal structure has revealed that a peptide (ERWGHDFIK, referred to as the C-9 peptide in this study) at the C-terminus of PaAP blocks the key active site of PaAP [52]. The released C-9 peptide can serve as a substrate for PaAP and also inhibit the activity of PaAP when present at high concentrations (over 1 mM) [52]. Therefore, we investigated the potential of the C-9 peptide to repress the transcription of *lasI*. The C-9 peptide was added to the culture of the PAO1 strain carrying plasmid reporters with the p*lasI*::*gfp* fusion (PAO1/p*lasI*::*gfp*). The growth curve indicated that 100 μM of the C-9 peptide did not affect the growth of PAO1 (Fig. 5C). After 12 h of growth, PAO1 treated with the C-9 peptide exhibited lower levels of *lasI* transcription compared to untreated PAO1 (Fig. 5D), suggesting that the C-9 peptide is one of the signaling molecules that mediates the regulation of PaAP on the expression of *lasI*. Although C-9 peptides repressed the transcription of *lasI*, they did not affect the transcription of the other three genes involved in the HSL-dependent QS regulation: *lasR*, *rhlI*, and *rhlR*. Additionally, we tested the C-18 peptide, which is cleaved from the C-terminus of PaAP, and the N-12 peptide, derived from its N-terminus (24th to 36th residue) (Fig. 5B), for their ability to repress *lasI* transcription. Both C-18 and N-12 were capable of repressing *lasI* transcription, similar to the effect of the C-9 peptide (Fig. 5E). All three of these short peptides are products of PaAP during its enzymatic activation, which explains why the enzymatic activity of PaAP is necessary for regulating Psl production (Fig. 2A and B). We then investigated whether the presence of PaAP was required for the regulation of *lasI* transcription by the short peptides. C-9, C-18, and N-12 peptides can repress *lasI* transcription in Δ*paaP* strains (Fig. 5F), suggesting that this inhibitory effect does not require the presence of PaAP, even though PaAP is the source of the peptides.

**Figure 6:**
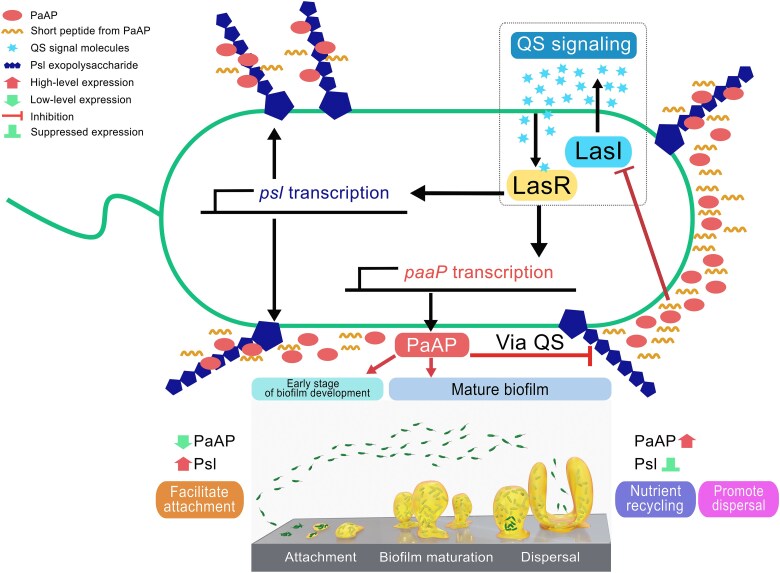
PaAP regulates the production of Psl exopolysaccharides through a feedback regulation on QS system. PaAP associates with Psl exopolysaccharides extracellularly to make sure that PaAP functions as a nutrient recycler in a zone close to Psl-producing cells. PaAP regulates the production of Psl in a QS-dependent manner during biofilm development. At the early stage of biofilm development where cell density is low, PaAP is expressed at a low level. Bacteria are able to produce Psl and attach to surfaces or to each other to start a biofilm. As biofilm matures, cell density is high and QS signaling molecules are accumulated. Both PaAP and Psl are highly expressed. However, PaAP limits QS signaling via short peptides cleaved from pro-PaAP and subsequently repressed *psl* transcription, to avoid over-production of Psl and to facilitate further biofilm seeding dispersal. The schematic diagram of the biofilm development at the bottom was adapted with the author’s permission [36].

Taken together, the data in this study indicate that the LasI-activated extracellular aminopeptidase PaAP has the ability to feedback regulate its own expression by inhibiting *lasI* expression through short peptides cleaved from pro-PaAP during its enzymatic activation. Meanwhile, the repression of *lasI* expression by PaAP affects the production of the key matrix exopolysaccharide, Psl, thereby further regulating biofilm formation (Fig. 6).

## Discussion

Both extracellular proteins and exopolysaccharides are major components of the bacterial biofilm matrix. However, little is known about whether and how these components interact. PaAP is one of the most abundant proteins in the matrix of *P. aeruginosa* biofilms [3]. Psl is a critical exopolysaccharide in the matrix that enables both nonmucoid and mucoid strains of *P. aeruginosa* to initiate and maintain biofilms. In this study, we demonstrate that PaAP associates with Psl and regulates Psl production at the transcriptional level through QS signaling molecules.

We previously reported that Psl is anchored to the bacterial surface and also forms a fiber-like matrix that enmeshes bacteria within a biofilm [36]. It has been revealed that Psl provides social benefits to populations within biofilms [53]. In this study, we demonstrate that PaAP is co-purified or enriched with Psl. Extracellular enzymes can be secreted through OMVs, and PaAP is also a major component of these vesicles [9, 10]. PaAP has been reported to participate in interactions with lung epithelial cells, facilitating the delivery of OMV contents, including virulence factors [10]. OMVs may play a role in PaAP’s association with Psl, as membrane-staining materials have been shown to co-localize with the Psl matrix [34]. PaAP digests extracellular proteins or peptides into amino acids, which can also be considered a public good in biofilms [20]. It has been proposed that PaAP may not be fully secreted and could remain partially associated with the cell envelope, ensuring that the digestion products are preferentially available to the producer [54]. Here, we hypothesize that there may be an alternative mechanism to achieve this goal: PaAP associates with the exopolysaccharide Psl to ensure that PaAP functions in proximity to the bacterial cells producing Psl.

As a key component of biofilm scaffolding, Psl plays a crucial role in determining the biofilm formation ability of *P. aeruginosa*, including initial attachment and subsequent biofilm development. In this study, we report that the extracellular protein, PaAP, constrains the production of this major exopolysaccharide, Psl, without affecting bacterial growth in liquid culture. The transcription of *psl* was higher in the Δ*paaP* strain compared to PAO1, in all the three tested media (Jensen’s, M63, and ASM) (Fig. 4D), Although the difference in Psl production between these two strains was only shown in Jensen’s medium but not in the other two media (Fig. 3B and data not shown). This is likely due to the nutrient availability in M63 and AMS medium that is not sufficient to support robust exopolysaccharide production, making it impossible to discern differences in Psl production between PAO1 and the Δ*paaP* strain. A previous study has demonstrated that in a co-culture system with A549 cell, the Psl/cellular biomass ratio in the PaAP-negative mutant was lower than in the wild-type strain, as observed under confocal microscopy [28]. We speculate that the differences between our findings and this one may be attributed to the different nutrient supplement, as well as the methods used to quantify Psl production.

We have further demonstrated that the control of Psl exopolysaccharides by PaAP is mediated through QS signaling molecule, LasI. The expression of PaAP is known to be induced by LasI/LasR system. Here, we discover that extracellular PaAP exhibits feedback regulation on the transcription of *lasI*, resulting in the inhibition of *psl* transcription. Our data suggest that PaAP functions as a QS-controlled regulator, balancing not only its own production but also the Psl production during biofilm development. In the early stages of biofilm development, bacteria are primarily isolated from one another, thus few QS signaling molecules are accumulated. PaAP is expressed at a low level, allowing bacteria to produce Psl, to attach to surfaces or to each other, ultimately leading to forming a biofilm. As the biofilms matures, QS signaling molecules are accumulated within the biofilm, thus PaAP is expressed at a high level, allowing nutrient recycling within a biofilm. Meanwhile, PaAP restricts the QS acceleration and limits the production of Psl, which facilitates further biofilm seeding dispersal (Fig. 6). We have also discovered that several short peptides (C-9, C-18, and N-12 peptide) cleaved from pro-PaAP play a role in interfering the transcription of *lasI*, explaining why it requires the enzymatic activity of PaAP to regulate Psl production. Further studies would be required to elucidate how extracellular peptides are sensed or pass through the membrane. In summary, our data reveal the importance of extracellular enzymes within biofilms and indicate that they play roles in regulating QS signal and polysaccharides production during biofilm development.

## Data Availability

The data underlying this article are available in the article.
